# Seeing Beyond the Expected: An Uncommon Case of Plateau Iris Syndrome in the Outpatient Setting

**DOI:** 10.7759/cureus.59575

**Published:** 2024-05-03

**Authors:** Tayyab Shakoor, Birkaran S Sadhar, Paarth Sharma, Chris Buzas

**Affiliations:** 1 Ophthalmology, Lake Erie College of Osteopathic Medicine, Erie, USA

**Keywords:** plateau iris syndrome, iridotomy, primary angle-closure glaucoma, argon laser iridoplasty, gonioscopy

## Abstract

Patients presenting with elevated intraocular pressures (IOPs) refractory to laser peripheral iridotomy should be suspected to have plateau iris syndrome (PIS). We present an uncommonly seen case of a 59-year-old female who presented with blurred vision, left-sided head pain, and IOPs Oculus Uterque (OU). Despite medical and laser peripheral iridotomy, left eye pain and elevated IOPs persisted, which led to a suspected diagnosis of PIS. The patient was subsequently treated by a glaucoma specialist who performed argon laser iridoplasty. Following this procedure alongside appropriate pharmacologic maintenance treatment, the patient’s symptoms and elevated IOPs were resolved. With proper management, irreversible blindness can be prevented in PIS.

## Introduction

Plateau iris was first used in 1958 to describe a specific anatomical arrangement of the iris and ciliary body which commonly leads to an elevated intraocular pressure (IOP) [1]. The mean age of diagnosis is 35 and the condition is more prevalent in females [2]. There also appears to be an increased prevalence of plateau iris in those with a family history [3]. The condition is characterized by the narrowing of the anterior chamber angle which results from the iris being positioned anteriorly on the ciliary body or the anterior displacement of the ciliary body [1]. This repositioning of the iris obstructs flow through the trabecular meshwork and consequently elevates the IOP. This can eventually lead to chronic angle closure glaucoma and permanent loss of vision [1]. Plateau iris syndrome (PIS) is identified by a consistently narrow angle despite a patent iridotomy [4]. Adequate diagnosis and treatment are essential for preventing vision loss in individuals with plateau iris. The preferred method for evaluating angle opening is gonioscopy, and the treatment of choice is argon laser peripheral iridoplasty [1]. Early identification and intervention play crucial roles in achieving a favorable prognosis for patients with this ocular condition. Here we present a case of a patient with refractory elevated IOPs and eye pain, who was subsequently diagnosed with PIS and successfully treated with iridoplasty.

## Case presentation

The patient was a 59-year-old female with a past medical history of diabetes mellitus, depression, hypertension, dry eye, and presbyopia who presented to the outpatient ophthalmology clinic with complaints of blurred vision Oculus Sinister (OS) and pain in the left side of her forehead that waxes and wanes with photosensitivity. The patient showed signs of possible glaucoma. Ocular examination displayed IOPs of 20 mmHg Oculus Dexter (OD) and 28 mmHg OS, narrow and quiet Oculus Uterque (OU) of the anterior chamber with a cup-to-disc ratio of 0.4 OU (Table 1). Based on PE, anatomic narrow angles were causing aqueous pathology. The risk of additional attacks and risk of blindness were discussed with the patient. The patient agreed to laser peripheral iridotomy (LPI) OU (OS>OD) and to return if symptoms worsen. The patient was initially started on timolol maleate BID OS, to reduce aqueous humor production, and instructed to avoid anticholinergics, anticonvulsants (IE topiramate), and antidepressants as they can exacerbate acute angle closure glaucoma. She underwent YAG LPI OS without complication and was instructed to follow up after a five-day tapered course of prednisolone to reduce inflammation. After completion of the steroid course, YAG LPI OD was completed without complication. Two months later, the patient called the office complaining of one week of unremitting severe OS eye pain that worsened after waking up on arrival. IOP was noted to be 23 mmHg OD and 49 mmHg OS, and the iridotomies were found to be patent (Table 1).

**Table 1:** Pre-operative and Post-operative Trending Eye Pressures YAG LPI: YAG Laser peripheral iridotomy; OD: Oculus Dexter; OS: Oculus Sinister; OU: Oculus Uterque; mmHG: Millimeter of Mercury

Pre/Post-operation Eye Pressures	Right Eye	Left Eye
Pre-operation	20 mmHG OD	28 mmHG OS
Two-Week Follow-Up YAG LPI OS	16 mmHG OD	16 mmHG OS
Two-Week Follow-Up YAG LPI OD	18 mmHG OD	21 mmHG OS
Two-Month Follow-Up YAG LPI OU	23 mmHG OD	49 mmHG OS
Four-Week Follow-Up Argon Laser OU	24 mmHG OD	16 mmHG OS
Two-Month Follow-Up Argon Laser OU	23 mmHG OD	24 mmHG OS
Six-Month Follow-Up Argon Laser OU	16 mmHG OD	13 mmHG OS

**Figure 1:**
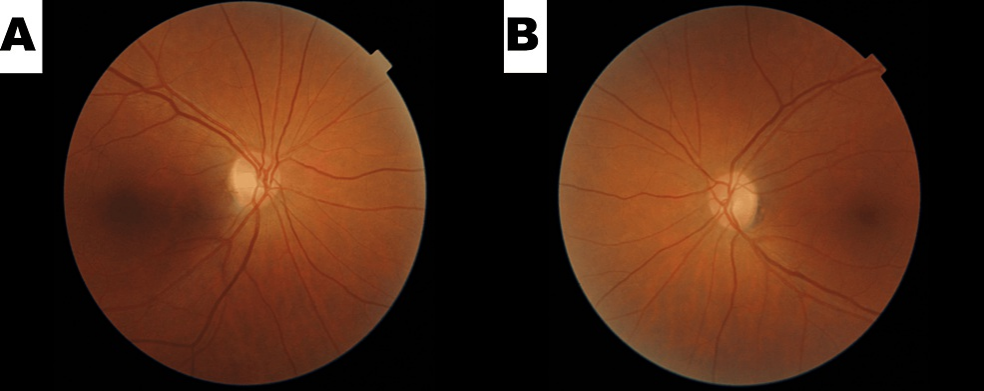
Fundoscopy of normal OD (Pane A). Fundoscopy of Normal OS (Pane B) Showing No Irreparable/Lasting Damage from Increased IOP OD: Oculus Dexter; OS: Oculus Sinister; IOP: Intraocular pressure

Treatment with both Simbrinza (brinzolamide/brimonidine) and Diamox (acetazolamide) reduced aqueous humor formation, while Rocklatan (netarsudil/latanoprost) increased aqueous humor outflow. When combined, the medications reduced the IOP to a normal pressure while the patient was still in the office. The patient was subsequently referred to a glaucoma specialist for possible PIS. Follow-up with the patient later revealed she had argon laser iridoplasty OU done with interval deepening of the anterior chamber. Her IOPs at subsequent visits were found to be controlled (Table 1). Follow-up fundus exam imaging revealed no residual damage or anatomic changes from the elevated IOP (Figure 1). The secondary laser effectively treated her angle-closure glaucoma, and she only required topical medical glaucoma therapy to keep her IOP at goal.

## Discussion

**Figure 2:**
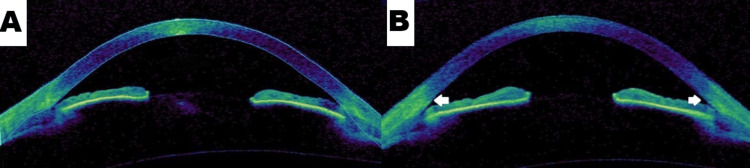
Anterior Segment OCT: Pre-LPI (Pane A) and Post-LPI (Pane B). Note the Widening of the Angle (White Arrows) OCT: Optical coherence tomography; LPI: Laser peripheral iridotomy. Plateau Iris. (2011). Accessed: February 20, 2024: https://webeye.ophth.uiowa.edu/eyeforum/cases/143-plateau-iris.htm. [6] Permission obtained from EyeRounds.org, University of Iowa (Rogers GM, Alward LMW, Fingert JH: EyeRounds.org - Ophthalmology - The University of Iowa)

First termed in 1958, plateau iris is a rare anatomic variant of primary-angle closure glaucoma that causes mechanical obstruction of the trabecular meshwork in young patients due to an abnormal ciliary body [1]. One small study concluded a 32% incidence of plateau iris after LPI on ultrasound biomicroscopy; however, there is no conclusive number on its exact incidence, only that it is uncommonly seen [5]. Diagnosed via optical coherence tomography or ultrasound biomicroscopy, this rare anatomic variant is distinct in its lack of response when patients undergo iridotomy, resulting in the need for argon laser peripheral iridoplasty (Figure 2) [4,6]. The typical demographic for PIS tends to be young (typically under the age of 60), female, and individuals of Caucasian descent with a family predisposition for angle-closure glaucoma [7]. The pathway of aqueous humor drainage in the eye begins in the posterior chamber through the pupil and into the trabecular meshwork where it inserts into the angle of the anterior chamber [8]. Abnormal positioning of the iris, caused by a larger and more anterior ciliary body or a shorter-than-normal iris root, can obstruct aqueous flow, leading to an elevation in IOP [9]. Clinical suspicion for plateau iris should arise when a patient experiences symptoms such as headaches, nausea, vomiting, blurry vision, and halos around lights, and this should be emergently investigated for acute angle closure glaucoma [7]. Physical examination with a slit lamp may show a normal anterior chamber depth with a flat iris surface. The gold standard for assessing the angle opening is gonioscopy which can illustrate a characteristic double hump sign [1]. Moreover, tonometry to measure IOP before and after iridotomy and dilation of the pupil can show a clinical picture of PIS alongside fundoscopy.

Additional testing with ultrasound biomicroscopy can display an anatomic variation of the ciliary body [10]. Some possible conditions to consider other than PIS include iridociliary cysts, pupillary block, peripheral anterior synechiae, lens-induced angle closure, and nanophthalmos to name a few [2]. The first step in treatment/management is primary prevention before symptom onset. This emphasizes the importance of screening those with a significant family history or those with first-degree family relations with plateau iris to avoid lasting consequences such as irreversible blindness. Medical management should promote aqueous outflow through the use of mitotic agents such as pilocarpine, carbachol, aceclidine, and dapiprazole. In addition, surgical management always begins with peripheral iridectomy/iridotomy which is diagnostic and therapeutic (Figure 2) [1,6]. If that procedure were to not provide definitive care, argon laser peripheral iridoplasty can widen the iridocorneal angle [11]. In refractory cases of PIS, treatments such as anterior chamber paracentesis, trabeculectomy, goniosynechialysis, lens extraction, and shunt surgery can be performed [12]. With post-op screenings, management of symptoms to assess for angle narrowing, and signs of glaucoma, prognosis tends to be favorable [1]. PIS does not need to be a syndrome that leads to irreversible blindness if screened and treated early.

## Conclusions

Although its incidence is widely unknown, PIS should be considered a differential for patients with refractory increased IOPs. Persistence of narrow-angle glaucoma following iridotomy warrants a diagnosis of PIS. This increased IOP can be managed further with miotic agents and argon laser peripheral iridoplasty. Moreover, eventual surgical management should be explored if the glaucoma is unresponsive. Due to its rarity of presentation along with its prevalence within younger patients, appropriate management with a glaucoma specialist should be established with continued follow-up to avoid permanent vision loss.

## References

[1] Hoyos CE, Ferreira MC, Libreros-Peña L, Shah MA, Aristizabal JC, Muñoz E, Seth A (2023). Plateau iris syndrome: epidemiology, diagnosis, and treatment: a narrative review. Oman J Ophthalmol.

[2] Ritch R, Chang BM, Liebmann JM (2003). Angle closure in younger patients. Ophthalmology.

[3] Etter JR, Affel EL, Rhee DJ (2006). High prevalence of plateau iris configuration in family members of patients with plateau iris syndrome. J Glaucoma.

[4] Stefan C, Iliescu DA, Batras M, Timaru CM, De Simone A (2015). Plateau iris--diagnosis and treatment. Rom J Ophthalmol.

[5] Kumar RS, Baskaran M, Chew PT (2008). Prevalence of plateau iris in primary angle closure suspects an ultrasound biomicroscopy study. Ophthalmology.

[6] Rogers GM, Alward LMW, Fingert JH (2024). Plateau Iris. https://webeye.ophth.uiowa.edu/eyeforum/cases/143-plateau-iris.htm.

[7] Stieger R, Kniestedt C, Sutter F, Bachmann LM, Stuermer J (2007). Prevalence of plateau iris syndrome in young patients with recurrent angle closure. Clin Exp Ophthalmol.

[8] Goel M, Picciani RG, Lee RK, Bhattacharya SK (2010). Aqueous humor dynamics: a review. Open Ophthalmol J.

[9] Llinas A, Dorairaj S, Liebmann JM, Ritch R (2008). Plateau iris syndrome in a child. Eye (Lond).

[10] Diniz Filho A, Cronemberger S, Mérula RV, Calixto N (2008). Plateau iris. Arq Bras Oftalmol.

[11] Bourdon H, Aragno V, Baudouin C, Labbé A (2019). Iridoplasty for plateau iris syndrome: a systematic review. BMJ Open Ophthalmol.

[12] Ng WT, Morgan W (2012). Mechanisms and treatment of primary angle closure: a review. Clin Exp Ophthalmol.

